# Photothermal foveal injury secondary to accidental alexandrite-laser exposure: a case report and literature review

**DOI:** 10.3389/fopht.2026.1948525

**Published:** 2026-10-28

**Authors:** Marya Allan, Sana’ Muhsen, Eman Yaseen, Nisreen Sawafta, Mousa Alfoqahaa

**Affiliations:** 1 Faculty of Medicine, Al-Quds University, Abu Dis, Palestine; 2 The University of Jordan School of Medicine, Amman, Jordan

**Keywords:** alexandrite laser, foveal laser injury, optical coherence tomography (OCT), retina, vision, visual field

## Abstract

**Purpose:**

To report a case of alexandrite laser-induced foveal injury complicated by acute anterior uveitis, which showed a significant clinical improvement over serial follow-up.

**Observations:**

We report the case of a 23-year-old female, a laser hair removal technician, who presented with sudden central visual loss in her left eye (LE) after an accidental exposure to an alexandrite laser beam while cleaning the probe. At the initial presentation, visual acuity (VA) of the LE was 0.1. Optical coherence tomography (OCT) demonstrated a loss of the foveal depression with a vertical hyperreflective column extending from the inner retinal layers to the retinal pigment epithelium (RPE), with focal disruption of the ellipsoid zone (EZ) and the external limiting membrane (ELM) at the fovea. Over 5 months with regular follow-up, VA improved to 1.0. OCT demonstrated residual central foveal disruption with localized attenuation of the EZ and RPE, confined to a smaller area compared with baseline.

**Conclusions and importance:**

This case demonstrates the importance of safety protocols when handling laser devices to prevent accidental eye injuries, and demonstrates the potential for good clinical outcomes with regular evaluation and follow-ups.

## 1 Introduction

Multiple laser devices have been developed as long-term hair removal methods, utilizing selective photothermolysis, in which specific wavelengths target melanin pigments in the hair follicle while sparing surrounding tissue (1). These include the long-pulse ruby (694 nm), long-pulse alexandrite (755 nm), long-pulse diode (810 nm), and long-pulse Nd : YAG (1,064 nm) lasers (1). While generally safe, these lasers have the potential to cause ocular injuries (2, 3).

An alexandrite laser uses an alexandrite crystal as its laser medium and is considered a red-light laser (4). Accidental direct eye exposure to the emitted light can lead to macular or retinal damage. Examples include laser-induced maculopathy and chorioretinal burns leading to vision loss, which are typically due to insufficient eye shielding, sudden gaze into the laser beam, or patient movement during facial treatment (5). Therefore, eye protection during these cosmetic and medical dermatological procedures is essential to prevent ocular complications and trauma.

## 2 Case description

A 23-year-old female presented urgently to our institution in April 2025 complaining of acute loss of vision in her left eye (OS) after accidental exposure to a laser beam. The patient works as a laser hair removal technician at a medical center. She reported being exposed to the laser beam by accident while cleaning the probe, after ending the laser session with her patient and after taking off the protective glasses less than an hour prior to presentation. The machine used was the GentleLase Pro™ manufactured by Candela Corporation, Wayland, Massachusetts (MA), USA. The settings used were as follows: spot size of 18 mm, fluence (energy) of 20 J/cm^2^ with a pulse duration of 20 ms. She reported an immediate black spot in the central field of her left eye. The patient had no previous ophthalmic history. On arrival, her unaided visual acuity (VA) was 1.0 in the right eye (OD) and 0.1 OS with no improvement with refraction. Amsler grid testing demonstrated an absolute central scotoma in the OS.

On day 1 after the injury, the anterior segment examination of the OD was within normal limits. OS showed a clear cornea, trace cells, sectoral heterochromia, previously unnoticed by the patient, and a clear lens. Intraocular pressure (IOP) was 14 mmHg in both eyes (OU). Dilated fundus examination showed a circular yellow (hypopigmented) lesion in the left fovea (**Figure 1**). Otherwise, the rest of the fundus examination was normal, and the fellow eye was normal. Optical coherence tomography (OCT) of the macula showed a disruption of the foveal contour with loss of foveal depression, and a foveal hyperreflective vertical column extending from the inner retinal layers down to the retinal pigment epithelium (RPE), consistent with focal photothermal retinal injury. Associated localized disruption of the ellipsoid zone (EZ) and external limiting membrane (ELM) with focal discontinuity at the fovea and minimal disruption of RPE/Bruch’s complex in the fovea was also noted (**Figure 1**). No significant intraretinal or subretinal fluid was detected. The surrounding macula appeared normal (**Figure 1**). Meanwhile, OCT angiography (OCTA) of the OS macula showed that the superficial retinal plexus had intact vascularity and a preserved foveal avascular zone (FAZ) architecture. The deep retinal plexus showed mild thinning of central capillaries and minimal attenuation of central flow signal. The outer retina showed a central hyporeflective signal loss. The choriocapillaris (CC) showed a well-demarcated, round central flow deficit at the fovea with a complete signal void. This finding corresponded to the lesion seen on the fundus photograph and OCT (**Figure 1**). The patient was advised to maintain close follow-up, and no medications were given at the initial presentation. The patient presented 2 days later, complaining of left eye pain, light sensitivity, and redness with no change in vision. Examination revealed left acute anterior uveitis in the OS (+2 cells) with normal IOP and no change in fundus examination. She was prescribed topical prednisolone acetate 1% eye drops every hourly.

**Figure 1:**
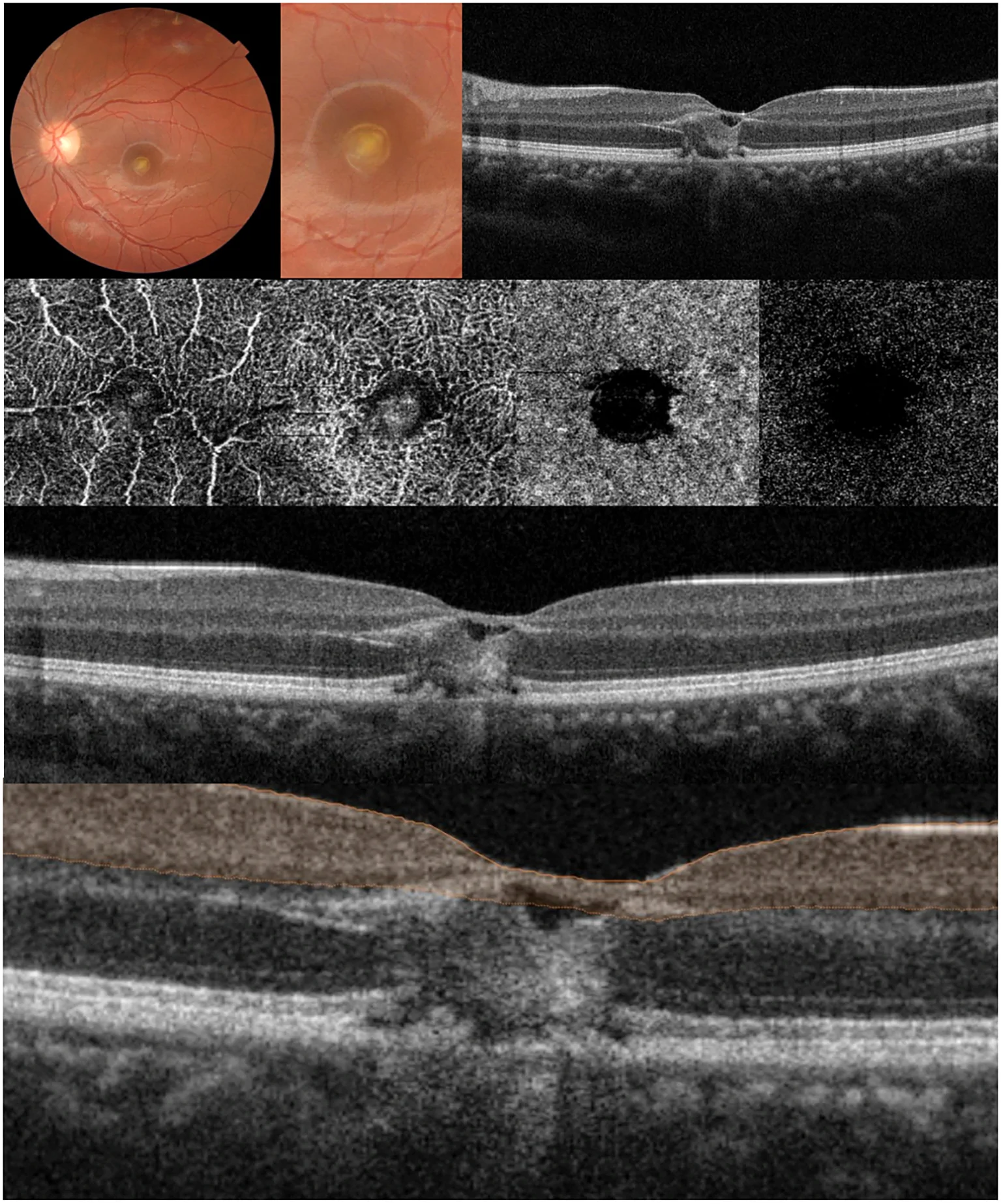
On 28/4/25, dilated fundus examination of the OS showed a circular yellow lesion in the fovea. Macular OCT of the OS showed loss of the foveal depression with a vertical hyperreflective column extending from the inner retina to the RPE, with focal disruption of the EZ and ELM at the fovea. Macular OCTA of the OS showed intact vascularity and a preserved FAZ, mild thinning of central capillaries, and minimal attenuation of central flow signal at the deep retinal plexus, with a central hyporeflective signal loss at the outer retina. CC demonstrated a central flow deficit at the fovea with a complete signal void.

At her one-week follow-up, the patient reported some improvement in vision and a decrease in the size of the central scotoma. She also felt less pain and better light tolerance. VA improved to 0.5 with resolution of cells. The Amsler grid showed a significant decrease in the size of the central scotoma. DFE showed a less bright lesion (**Figure 2A**). OCT showed significant improvement with a more localized discontinuity and irregularity of the EZ (IS/OS junction) at the foveal center (**Figure 2B**). A decrease in the size of the focal hyperreflective area extending from the outer retinal layers toward the RPE was also noted (**Figure 2B**). Topical steroids were tapered and stopped over 1 month.

**Figure 2:**
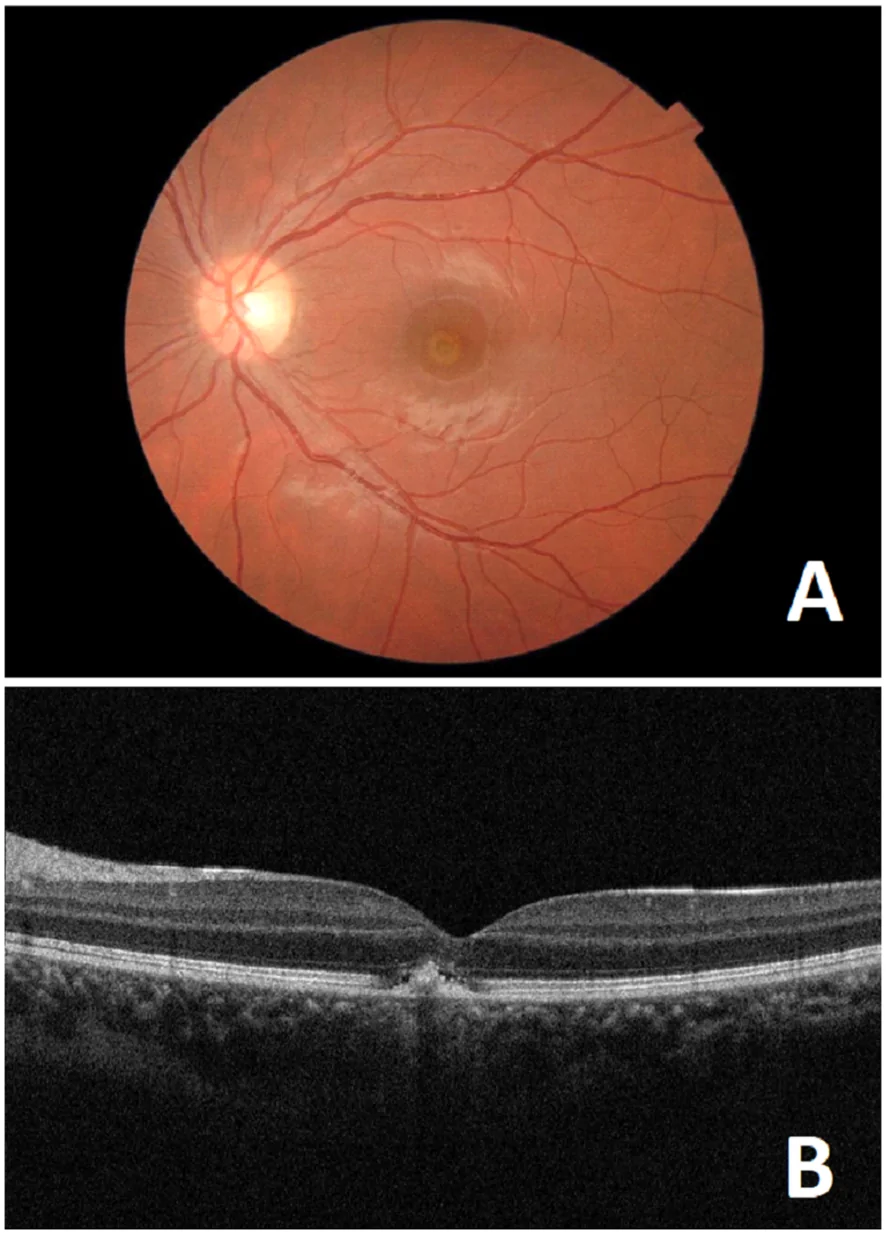
At the one-week follow-up, left eye fundus examination showed a less bright lesion **(A)**. OCT showed significant improvement, a decrease in the size of the focal hyperreflective area, and more localized discontinuity and irregularity of the ellipsoid zone (IS/OS junction) at the foveal center **(B)**.

At her final follow-up examination in September 2025, >4 months after the injury, the patient reported significant improvement in her vision. She described the residual central field defect as a pinpoint black dot in the center that she saw only when covering the right eye. Unaided VA of both OD and OS was 1.0. The heterochromia persisted, and the anterior chamber was quiet. IOP remained normal at 12 and 10 mmHg, respectively. DFE showed left foveal pigmentary mottling (**Figure 3**). OCT showed a disrupted central foveal contour with irregular reflectivity of the inner retinal layers. The outer retinal layers, especially the EZ and RPE, appeared attenuated and discontinuous, suggestive of persistent photoreceptor damage. All changes were confined to a significantly smaller area in comparison with the baseline OCT (**Figure 3**). OCTA of the macula OS compared with baseline OCTA showed that the most notable change was in the CC. Small and irregular hyporeflective foci at the fovea with partial return of underlying CC flow signal were noted. This change was consistent with resolution of overlying blockage and RPE clumping rather than true persistent CC dropout (**Figure 3**). The superficial and deep capillary plexuses remained largely intact with stable FAZ. The deep plexus showed improved central perfusion compared to baseline with the mild attenuation (**Figure 3**). The interval evolution from the acute to the atrophic stage highlighted overall significant anatomic improvement with transition to focal outer retinal atrophy/RPE scar, explaining persistent central scotoma but with reduced shadowing artifact on the CC slab.

**Figure 3:**
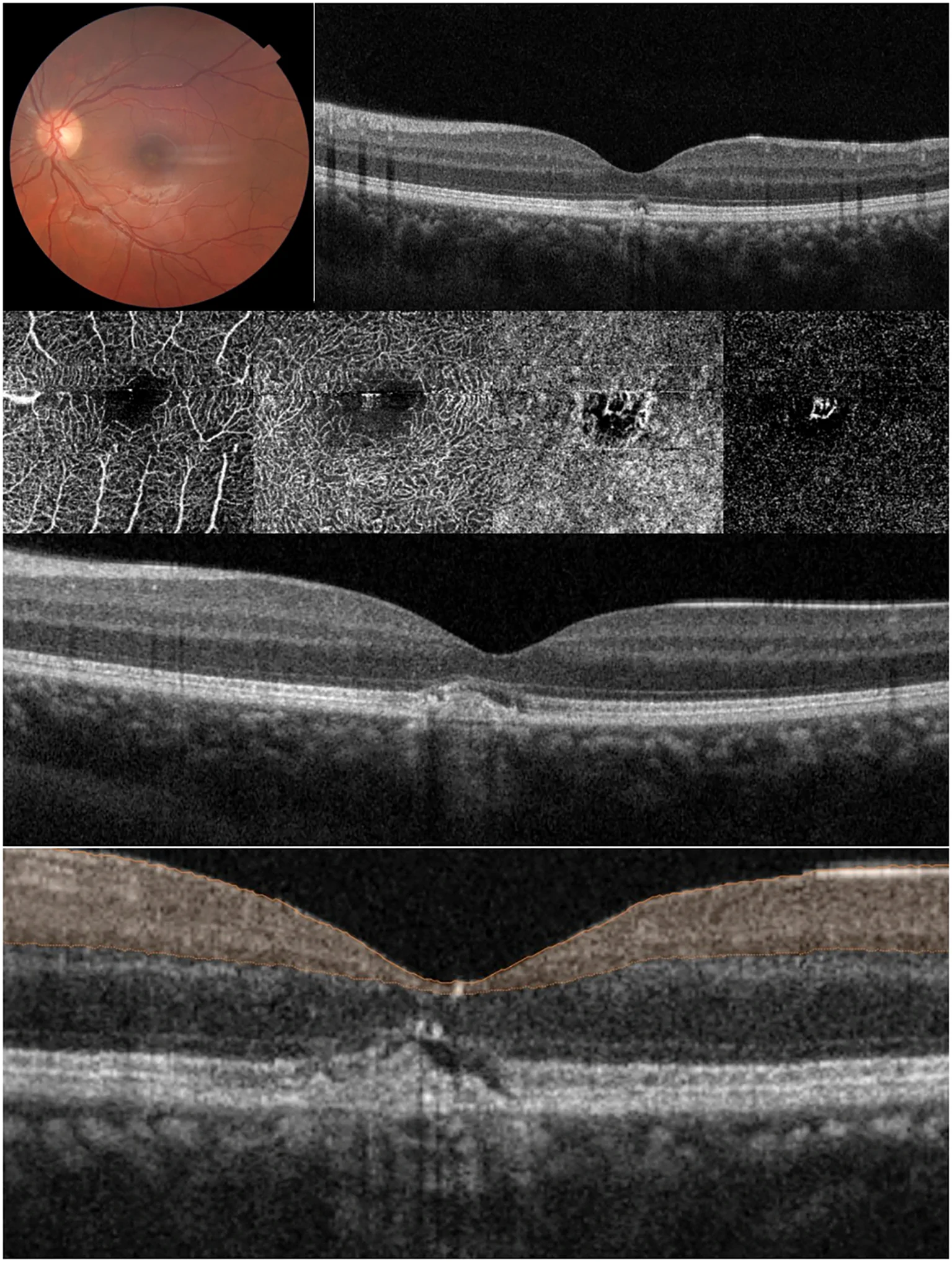
On 2/9/2025, fundus examination showed left foveal pigmentary mottling. OCT showed persistent photoreceptor damage. Macular OCTA of the OS, compared with baseline OCTA indicated a partial return in CC flow signal, presented as small and irregular hyporeflective foci at the fovea. The superficial and deep capillary plexus remained largely intact, with a stable FAZ and improved central perfusion of the deep plexus.

A visual field test was performed at her final visit (September 2025). The OS showed mild central visual field depression, consistent with trauma to the foveal area. On 24–2 testing, this appeared as generalized mild central depression (**Figure 4**), while the more sensitive 10–2 test detected small focal areas of dysfunction in the central 10°, particularly inferotemporal to fixation. Peripheral fields were preserved.

**Figure 4:**
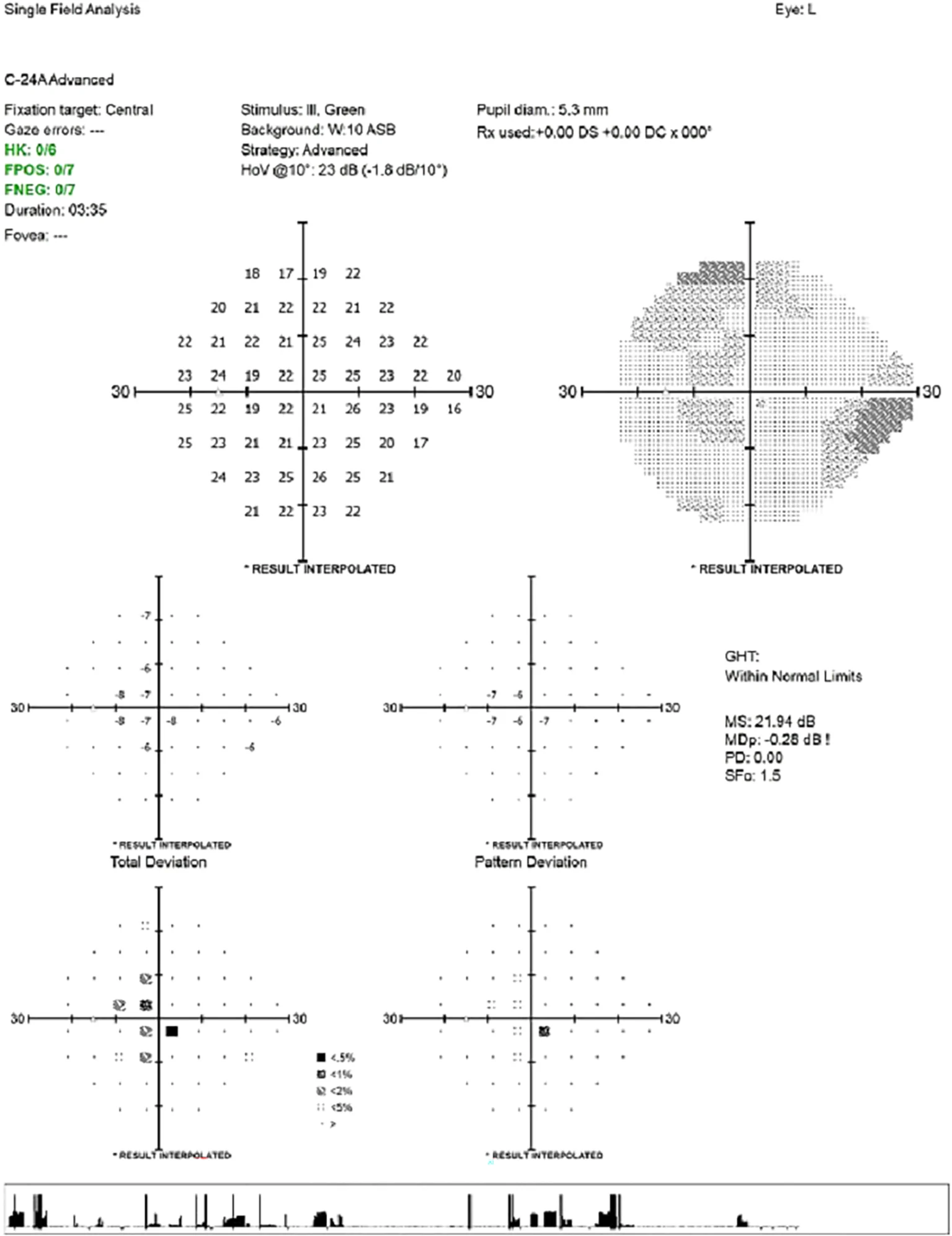
Left eye visual field. The 24–2 test shows generalized mild central depression.

## 3 Discussion

The incidence of laser-induced eye injuries is increasing over time due to the increased use of laser devices for hair removal and other cosmetic procedures, as well as the widespread availability of handheld laser devices and toy lasers, their low cost, and children’s easy access to them. These injuries range from mild to severe vision-threatening injuries (6, 14). The retina is the most vulnerable tissue to laser-induced injury (2), and injury from accidental laser exposure is becoming common due to inadequate eye protection during hair-removal procedures and can occur through photothermal, photochemical, and photomechanical pathways (8).

Alexandrite (755 nm) lasers, commonly used in cosmetic hair removal, can harm vision when unprotected eyes are exposed (2, 9, 10). The severity depends on exposure duration, wavelength, amount of laser exposure, and proximity to the fovea (2). Complications include burns of the cornea, iris atrophy, uveitis, cataract, and retinal burns, which vary in their presenting symptoms (including pain, photophobia, conjunctival hyperemia, blurry vision, and visual field defects) (2). Several case reports describe these harmful effects, including acute foveal and parafoveal burns (11), which may result in visual impairment that varies in severity. Although some lesions may improve over time, others can result in irreversible damage and visual deficits (2). Delayed complications such as choroidal neovascularization (CNV), may also develop days to years after exposure (10, 12).

One reported case described an aesthetician presenting after unprotected eye exposure to an alexandrite laser, complaining of a visual field defect, photophobia, and pain in the OS. FE showed a yellowish spot in the central fovea (2), representing acute retinal injury and disruption of the outer retina (13). VA declined to 0.32 (2). OCT demonstrated hyperreflective changes, but intact RPE and preserved EZ; no retinal disruption, intraretinal/subretinal fluid, ischemia, or choroidal abnormalities were observed. Despite management with topical steroids, cyclopentolate, and non-steroidal anti-inflammatory drugs (NSAIDs), there was significant damage to VA and no improvement in FE (2). In contrast, other cases demonstrated visual recovery depending on the location and severity of the lesion (9, 10).

Two significant cases highlighted complicated CNV after alexandrite exposure and reported a good prognosis, recovery of VA, and complete regression of CNV with no recurrence, following intravitreal injections of bevacizumab (9) and aflibercept (7) intravitreal injections.

Given that delayed complications such as CNV may occur days to years after exposure, patients should be clearly advised to monitor their vision and seek urgent medical attention if they notice any changes in their vision.

## 4 Conclusion

Eye injuries due to sudden laser exposure can either irreversibly affect vision, either directly by photoreceptor injury or indirectly by later complications such as CNV, or cause reversible or minimal field loss, as in this case. The common denominator among these cases was the lack of adequate protection when dealing with cosmetic lasers. Therefore, it is essential to adhere to proper eye protection protocols during such procedures, and for beauty centers to be under strict oversight from relevant authorities to ensure the implementation of safety standards and procedures, thereby enhancing the safety of patients and the aesthetician teams that handle them.

## Data Availability

The original contributions presented in the study are included in the article/supplementary material. Further inquiries can be directed to the corresponding author.
